# Apolipophorin-II/I Contributes to Cuticular Hydrocarbon Transport and Cuticle Barrier Construction in *Locusta migratoria*

**DOI:** 10.3389/fphys.2020.00790

**Published:** 2020-07-08

**Authors:** Yiyan Zhao, Weimin Liu, Xiaoming Zhao, Zhitao Yu, Hongfang Guo, Yang Yang, Jianqin Zhang, Bernard Moussian, Jianzhen Zhang

**Affiliations:** ^1^Research Institute of Applied Biology, Shanxi University, Taiyuan, China; ^2^College of Life Science, Shanxi University, Taiyuan, China; ^3^Modern Research Center for Traditional Chinese Medicine, Shanxi University, Taiyuan, China; ^4^Interfaculty Institute of Cell Biology, University of Tübingen, Tübingen, Germany; ^5^Université Côte d’Azur, CNRS, Inserm, iBV, Nice, France

**Keywords:** *Locusta migratoria*, cuticular lipids, apolipophorin, cuticular hydrocarbons, RNAi

## Abstract

Apolipophorins are carrier proteins that bind lipids and mediate their transport from tissue to tissue in animals. Apolipophorin I and II (apoLp-II/I) are the major apolipophorins in insects. The implication of apoLp-II/I in cuticle lipid-barrier formation in insects has not been addressed to date. In the present study, we investigated the function of apoLp-II/I in the migratory locust *Locusta migratoria* (*LmapoLp-II/I*). During the development of fifth instar nymphs, *LmapoLp-II/I* transcript levels increased until mid-instar, and then decreased gradually until molting to the adult stage. We found that *LmapoLp-II/I* was predominately expressed in the fat body and the integument including oenocytes and epidermal cells. Immunodetection experiments revealed that LmapoLp-I mainly localized in the cytoplasm of oenocytes and epidermal cells. Silencing of *LmapoLp-II/I* caused molting defects in nymphs. Importantly, RNA interference against *LmapoLp-II/I* resulted in a significant decrease in the content of cuticle surface lipids including alkanes and methyl alkanes. Cuticular permeability was significantly enhanced in these nymphs in Eosin Y penetration assays. By consequence, desiccation resistance and insecticide tolerance of ds*LmapoLp-II/I*-treated locusts were reduced. Taken together, our results indicate that *LmapoLp-II/I* is involved in the transport and deposition of surface-cuticular lipids that are crucial for maintaining normal cuticle barrier function in *L. migratoria*.

## Introduction

Apolipoproteins are carrier proteins that bind lipids to form lipoprotein particles called lipophorins. In mammals, the major apolipoproteins (apo) are generally divided into four categories including apoA, apoB, apoC, and apoE (Mahley et al., 1984; Liu et al., 2019). These proteins are important components of the blood plasma, and mediate trafficking of various lipids between producing and consuming organs (Su and Peng, 2020). In insects, apolipoproteins were referred to as apolipophorins (apoLp) (Beenakkers et al., 1981). Apolipophorins are the main components of insect lipophorin particles. Lipophorin (Lp) was isolated and purified from the hemolymph of various insects such as *Hyalophora cecropia, Philosamia cynthia, Periplaneta americana* and *Locusta migratoria* (Thomas and Gilbert, 1968; Chino et al., 1969, 1981; Chino and Kitazawa, 1981), as a reusable shuttle, whose major role is transport of lipids including diacylglycerol, phospholipids, sterols, and hydrocarbons between tissues (Ryan and Van Der Horst, 2000).

The insect lipophorin is composed of two non-exchangeable apolipophorins, apolipophorin I (apoLp-I, ∼240 kDa) and apolipophorin II (apoLp-II, ∼80 kDa), and may additionally contain an exchangeable protein, apolipophorin III (apoLp-III, ∼18 kDa) (Ryan and Van Der Horst, 2000). ApoLp-I and apoLp-II are derived from a common precursor protein, apolipophorin II/I (apoLp-II/I) through post-translational cleavage (Weers et al., 1993). ApoLp-II/I is a homolog of the mammalian apoB and belongs to the same superfamily of large lipid transfer proteins (LLTP) (Van Der Horst and Rodenburg, 2010), while apoLp-III is homologous to mammalian apoE (Weers and Ryan, 2006).

Molecular work on apolipophorins was initiated in *Manduca sexta* and *L. migratoria*. The cDNA sequences of *apoLp-III* were first cloned from these two insect species (Cole et al., 1987; Kanost et al., 1988). The biochemical and molecular properties of apoLp-III and apoLp-II/I were explored in locusts (Van Antwerpen et al., 1988; Van Der Horst et al., 1991; Weers et al., 1993), which are involved in purification of apoLp-III, origination of apoLp-II and I and immunocytochemical localization of all of them. Subsequently, it was reported that locust *apoLp-II/I* was strongly expressed in pigmented glial cells of the lamina underlying the locust retina (Bogerd et al., 2000). It localized to the basement membrane suggesting an implication of apoLp-II/I in the transport of retinoids and/or fatty acids to the insect retina. Interestingly, it was shown that apoLp-III replaced apoLp-II/I in high density lipophorin (HDLp) to recruit more DAGs, resulting in the transformation of HDLp to low density lipophorin (LDLp) particles during the prolonged flight of locusts (Van Der Horst and Rodenburg, 2010). In addition, apoLp-III also participated at an innate immunity response to microorganism infection in many insects (Zdybicka-Barabas and Cytryńska, 2013). Compared to apoLp-III, there are fewer studies on the function of apoLp-II/I. In the tsetse fly *Glossina morsitans morsitans*, RNA interference (RNAi) against *GmmapoLp-II/I* resulted in decreased hemolymph lipid levels in females and delayed oocyte development (Benoit et al., 2011). In the fruit fly *Drosophila melanogaster*, DmapoLp-II/I was shown to be the major lipid transport protein in the larval hemolymph (Palm et al., 2012). It was expressed in the fat body, secreted to the hemolymph and tethered to the gut, where it took up lipids and delivered them to consuming tissues such as imaginal discs and the brain. Although the roles of apoLp as circulating components of the hemolymph are well established, their functions in lipid trafficking to the cuticle surface in insects are largely unknown.

Cuticular lipids of insects are usually found on the cuticle surface and are composed of hydrocarbons (saturated alkanes and unsaturated alkenes), fatty acids, fatty alcohols, and wax esters (Chapman, 2013). In insects, cuticular lipids provide protection against water loss and prevent penetration of xenobiotics (Howard and Blomquist, 2005; Balabanidou et al., 2016, 2019; Wen et al., 2016). As reported, Lp serves as a carrier for the transport of hydrocarbons from the site of synthesis (oenocyte) to the site of deposition (cuticle) in *P. americana* and *L. migratoria* (Katase and Chino, 1982, 1984). It is speculated that after synthesis in the oenocytes, the lipids bind to apoLps and release into the hemolymph, subsequently shuttle to the epidermis where they bind to lipoprotein receptors, and finally transported to the cuticle surface via pore canals (Chapman, 2013). However, our understanding of how apoLps affect the molecular processes of the deposition of surface-cuticular lipids as well as the cuticular lipids dependent cuticle barrier construction in insects remains fragmentary.

In the present study, we analyzed the function of apoLp-II/I in *L. migratoria* (LmapoLp-II/I) in an RNAi-based approach. We found that *LmapoLp-II/I* was essential for the molting of locusts. We also showed that the deposition of cuticular lipids was dependent on LmapoLp-II/I. Both the inward and the outward barrier functions of the cuticle were compromised in *LmapoLp-II/I*-knockdown animals. The migratory locust *L. migratoria* is an important worldwide agricultural pest and has strong adaptability to high temperature and desiccation. This work adds new knowledge to the current understanding of apoLp-II/I function in insects and identifies apoLp-II/I as a potential target for pest management.

## Materials and Methods

### Insect Rearing

The eggs of *L. migratoria* were purchased from Insect Protein Co., Ltd., Cangzhou City, China. They were incubated in a climate chamber at 30 ± 2°C and 40 ± 10% relative humidity (RH). After hatching, the first instar nymphs were transferred to a gauze cage and fed with fresh wheat sprouts in a 14:10-h light: dark photoperiod. Fresh wheat sprouts were added daily until the nymphs grew to the third instar, thereafter, fresh wheat was supplemented with wheat bran.

### Bioinformatics Analysis of *LmapoLp-II/I*

The cDNA sequence of *LmapoLp-II/I* was obtained from the NCBI database. It is identical with one identified previously by Bogerd et al. (2000). The amino acid sequence of LmapoLp-II/I was translated from the cDNA sequence by the translation tools at ExPASy^1^. Protein domains were analyzed using SMART^2^. Protein domain composition was drawn using the Adobe Illustrator CS6 software (Adobe, United States). The molecular weight and isoelectric point were predicted using software at the EXPASY proteomics server^3^.

### Construction of Phylogenetic Tree

A phylogenetic analysis of apoLp-II/I was performed by using the full-length amino acid sequences of representative apoLp-II/I proteins in different insects such as *Blattella germanica*, *D. Melanogaster*, and *Zootermopsis nevadensis* retrieved from GenBank database. As described previously (Zhao et al., 2018), the phylogenetic tree was constructed with the MEGA 5.1 software based on a multiple alignment of the amino acid sequences performed by Clustal W, and by adopting the neighbor-joining (NJ) method. A bootstrap analysis of 1000 replications was performed, and 50% cut-off bootstrap values were used to condense the tree. The GenBank accession numbers of apoLp-II/I from the different species used to construct the phylogenetic tree are listed in Supplementary Table S1.

### Tissue-Specific and Developmental Expression Analysis

To analyze the tissue-specific and developmental expression patterns of *LmapoLp-II/I*, eleven tissues including integument, wing pads, foregut, gastric caeca, midgut, hindgut, Malpighian tubules, hemolymph, fat body, testis and ovary were dissected from 2-day-old fifth-instar nymphs, and the integument from 1-day-old fifth-instar nymphs (N5D1) to 8-day-old fifth-instar nymphs (N5D8) were collected. All samples were collected with four biological replicates, each with three nymphs. RNAiso Plus (TaKaRa, Japan) was used to extract total RNA of each samples according to the manufacturer’s recommendation. As described previously (Yu R. et al., 2016), one microgram of the total RNA was used to synthesize the first-strand cDNA using M-MLV reverse transcriptase (TaKaRa, Japan). Each cDNA sample was diluted 10-fold for reverse-transcription quantitative PCR (RT-qPCR) analysis. RT-qPCR was performed using SYBR^®^ Premix ExTaq^TM^ II (TaKaRa, Japan) on the ABI 7300 Real-Time PCR System (Applied Biosystems, United States). Two technical repetitions were made for each sample. Relative mRNA levels of target genes were calculated with the 2^–Δ^ ^*Ct*^ method, and the target gene expression level was normalized to the expression of the internal marker gene *EF1*-α that exhibited the most stable expression at different stages and in different tissues (Yang et al., 2014).

### RNA Interference

Specific primers for synthesizing double-stranded RNA (dsRNA) of *LmapoLp-II/I* (ds*LmapoLp-II/I*) and *GFP* (ds*GFP*) were designed at the E-RNAi web service^4^, which are listed in Supplementary Table S2. The ds*LmapoLp-II/I* and ds*GFP* were synthesized *in vitro* using T7 RiboMAX^TM^ Express RNAi System (Promega, United States) as described previously (Zhao et al., 2018). Thirty nymphs from 2-day-old fourth-instar (N4D2) were randomly selected for dsRNA injection. Each nymph was injected with 10 μg dsRNA, and the control group was injected with the same amount of ds*GFP*. At least three repetitions were performed. The injected locusts were raised under the same feeding condition. To determine silencing efficiency, different phenotypes of ds*LmapoLp-II/I-* or ds*GFP*-injected nymphs were collected for RT-qPCR analysis.

### Immunohistochemistry

To analyze the localization of LmapoLp-II/I protein, immunohistochemistry was performed as described previously (Yu R. et al., 2016). In brief, paraffin sections (5 microns thick) of the third abdominal integuments from 2-day-old fifth-instar nymphs after injection of ds*GFP* and ds*LmapoLp-II/I* were prepared. The polyclonal antibody against LmapoLp-I (LVAKRDIKSPDDYELK) was produced in mouse by the China Peptides Co., Ltd. (Shanghai, China). The mouse antiserum against LmapoLp-I (1:200) is used as a primary antibody at 4°C overnight to detect LmapoLp-I protein. After incubation with antibody, the sections were washed with PBS three times for 5 min each and then incubated with the Anti-mouse Alexa Fluor^®^ 594 (1:200, Thermo Fisher Scientific, United States) as secondary antibody for 2 h at 37°C. After washing three times for 5 min each with PBS, sections were incubated with Fluorescent Brightener 28 (FB28, 1 mg/ml, Sigma) for 5 s to detect chitin. The sample sections were then washed with PBS three times followed by incubated with SYTOX^TM^ Green Nucleic Acid Stain (Thermo Fisher Scientific, United States), which was diluted in PBS to a concentration of 0.025 μg/μl, for 15 min at room temperature to detect nucleus. The preimmune mouse serum was used as negative control. Images were captured using an LSM 880 confocal laser-scanning microscope (Zeiss, Germany) with excitation at 405 nm (FB28), 594 nm (Anti-mouse Alexa Fluor^®^ 594) and 488 nm (SYTOX^TM^ Green).

### Cryo-Sectioning and BODIPY Staining

The 3rd–5th abdominal segments of nymphs with different phenotypes (before ecdysis, during ecdysis and after ecdysis) after ds*LmapoLp-II/I* or ds*GFP* injection in 2-day-old fourth-instar nymphs were prepared for cryo-sectioning, respectively. The samples were dehydrated in a PBS containing increasing sucrose concentrations of 10, 20, and 30% (w/v), and then were embedded in the optimal cutting temperature compound (SAKURA, United States). Sections of 20 microns were prepared on a cryostat microtome (Leica, CM1950) at −25°C and blotted on SuperFrost Plus adhesion slides. The samples were baked on slide drier at 45°C for 3–4 h for tissue adhesion. The Bodipy staining was performed according to Yu et al. (2017). In brief, the sections were washed with PBS three times for 5 min each and then stained with Bodipy^505/515^ diluted in dimethyl sulfoxide to a concentration of 0.025 μg/μl (GE Healthcare, Germany) by incubation at 37°C for 30–45 min. After washing with PBS three times for 5 min each, the sections were stained with DAPI for 5 min at room temperature to label the nuclei. Images were observed and captured using an LSM 880 confocal laser-scanning microscope (Zeiss, Germany).

### Extraction and Quantification of Cuticular Hydrocarbons and Internal Hydrocarbons

After injection of ds*LmapoLp-II/I* or ds*GFP* in 2-day-old fourth-instar nymphs (N4D2), the nymphs at 48 h after dsRNA treatment (N4D4) and at 5 h after molting to the next stage (N5D1) were collected to quantify cuticular hydrocarbons (CHCs) and internal hydrocarbons (IHCs). Each nymph was immersed in 3 ml of *n*-hexane for 2 min before transferring the solvent to another chromatography bottle. This step was repeated twice. The three hexane extracts were combined and subjected to CHC purification and analysis by gas chromatography-mass spectrometry (GC-MS) on a TRACE 1310 coupled to an ISQ single-quadrupole MS detector with Xcalibur 2.2 software (Thermo Fisher Scientific, United States) as described previously (Yu Z. et al., 2016). The nymphs after the extraction of the CHCs were subjected to the extraction of IHCs. The wing pads, legs, guts and gonads were removed before extraction to exclude the effects of other factors, followed by extraction of IHCs with a mixture of *n*-hexane, methanol and water (2:1:1). After centrifugation, the *n*-hexane phase (supernatant) was recovered and purified as described previously (Yu Z. et al., 2016). Some of the alkanes were identified by their retention times compared to those of known standards (C7–C40 saturated alkanes Std, SUPELCO). The remaining alkanes were identified by Kovats retention index (I), which was calculated by an equation using retention time. These alkanes were quantified by their peak areas compared to that of the internal standard.

### Eosin Y Staining

Nymphs with different phenotypes (before ecdysis, during ecdysis and after ecdysis) after ds*LmapoLp-II/I* or ds*GFP* injection in 2-day-old fourth-instar nymphs were collected for Eosin Y staining to investigate the effect of cuticular lipids on cuticle permeability. The nymphs were transferred to a 2 ml microcentrifuge tube containing 1.5 ml of dye solution [0.5% eosin (W/V, Sigma, a red water-soluble dye with a molecular mass of 691.86 Da)], incubated at 45°C for 30 min, and then washed three times with water as described previously (Yu et al., 2017). Finally, the locusts were photographed with an Epson perfection V700 photo using the Epson Scan software.

### Desiccation Experiment

After injection of ds*LmapoLp-II/I* or ds*GFP* to 2-day-old fourth-instar nymphs (N4D2) for 24 h, nymphs were used for the desiccation experiment which were randomly divided into three groups and separately placed in a 50 ml bottle that covered with gauze and contained 2 ± 0.5 g of dry wheat bran at the bottom of the bottle for feeding. All test bottles were then placed in constant climate chamber (HPP110, Memmert, Germany) to control RH at 10 ± 2% for desiccation treatment and 50 ± 2% for control. The number of surviving insects was recorded every 12 h after desiccation treatment and the survival rate was calculated. The median lethal times (LT_50_) were obtained from a regression-probit value by using the SPSS program (SPSS Inc., Chicago, IL, United States). The corrected data were calculated by subtracting the effect at 50% RH from the effect at 10% RH. Overall survival was analyzed by the Kaplan–Meier method combined with the log-rank test. Three biological replicates were set up, and ten locusts were taken for each biological replicate.

### Bioassays With Insecticides After RNAi

To determine the effect of lipid changes on insecticide susceptibility, three different insecticide classes, organophosphate (malathion), carbamate (carbaryl), and pyrethroid (deltamethrin) were applied with topical application method (Zhang et al., 2017, 2019). The three insecticides were separately dissolved in acetone, and 3 μL of the solution (malathion for 120 μg/mL, carbaryl for 70 μg/mL, and deltamethrin for 8 μg/mL) was dropped on the abdomen of the locusts at 24 h after ds*LmapoLp-II/I* or ds*GFP* injection into 2-day-old third-instar nymphs that have the appropriate size for toxicology experiments. Mortality was determined at 24 h after insecticide exposure. Mean and standard errors for each group were performed in five independent bioassays, each with 11–13 nymphs.

### Expression Levels of Lipid Synthesis Related Genes After *LmapoLp-II/I* RNAi

To determine the effect of *LmapoLp-II/I* RNAi on lipid synthesis, the expression of lipid synthesis related genes, including acetyl-CoA Carboxylase (*LmACC*), fatty acid synthases (*LmFAS1, LmFAS2, and LmFAS3*), fatty acid elongases (*LmELO1-7*), and Cytochrome P450s (*LmCYP4G62* and *LmCYP4G102*) identified in our lab, was determined in the integument at 24 h after injection of ds*LmapoLp-II/I* or ds*GFP* on 2-day-old fourth-instar nymphs by RT-qPCR as described above. The primers used for RT-qPCR are listed in Supplementary Table S2.

### Statistical Analysis

The one-way analysis of variance test of the SPSS software (SPSS Inc., Chicago, IL, United States) was applied to analyze differences between different developmental stages and different tissues followed by the Tukey’s test. The Student’s *t-*test was carried out for silencing efficiency, CHCs quantity, LT_50_ and survival time with desiccation treatment, and mortality rate with insecticide treatment after RNAi. Asterisks indicate significant differences (^∗^*P* < 0.05; ^∗∗^*P* < 0.01; ^∗∗∗^*P* < 0.001).

## Results

### Bioinformatics and Phylogenetic Analysis of *LmapoLp-II/I*

The coding sequence of *LmapoLp-II/I* is 10,143 bp long and encodes a protein of 3380 amino acids containing a signal peptide, a lipoprotein N-terminal domain (LPD_N), a von Willebrand factor (vWF) type D (VWD) domain, a C8 domain and two domains (DUF1943 and DUF1081) with unknown function (Supplementary Figure S1A). This protein has a theoretical molecular weight of 371.8 kD and a pI of 6.18. Phylogenetic analysis using full-length amino acid sequences from other insect species revealed that LmapoLp-II/I clustered with the orthologs of *Blattaria* (Supplementary Figure S1B).

### Tissue-Specific and Developmental Expression Analysis of *LmapoLp-II/I*

**FIGURE 1:**
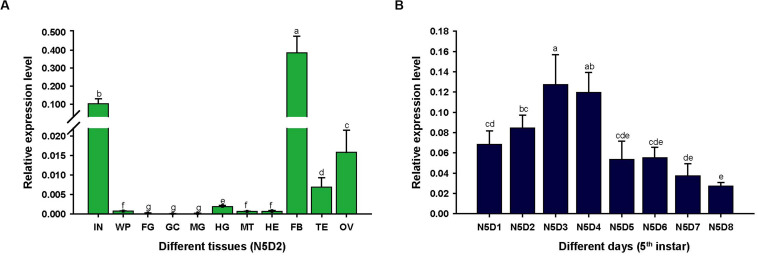
The relative expression levels of *LmapoLp-II/I* in different tissues and stages of the fifth instar nymphs in *L. migratoria* by RT-qPCR. **(A)** Expression of *LmapoLp-II/I* in different tissues of 2-day-old fifth-instar nymphs (N5D2) detected by RT-qPCR. The tissues are integument (IN), wing pads (WP), foregut (FG), gastric caeca (GC), midgut (MG), hindgut (HG), Malpighian tubules (MT), hemolymph (HE), fat body (FB), testis (TE), and ovary (OV). **(B)** Developmental expression profile of *LmapoLp-II/I* in the integument of N5D1-N5D8; *EF1-*α was used as the reference control. All data are reported as means ± SD of four independent biological replications. Different letters on the bars indicate significant difference among different samples (*P* < 0.05, Tukey’s HSD test; *n* = 4).

To assess the expression characteristics of *LmapoLp-II/I*, we analyzed the transcript levels of *LmapoLp-II/I* in different tissues and different developmental stages of fifth instar nymphs by RT-qPCR (Figure 1). *LmapoLp-II/I* transcripts were predominately detected in the integument and the fat body of 2 days old fifth instar nymphs (Figure 1A). Analysis of the *LmapoLp-II/I* temporal expression profile in the integument of fifth instar nymphs revealed that *LmapoLp-II/I* transcript levels increased in the first two days of this stage (N5D1-N5D2), reached a peak in 3 and 4 days old fifth instar nymphs (N5D3–N5D4), then decreased gradually until molting to the next stage (N5D5–N5D8) (Figure 1B).

### LmapoLp-I Localized to Epidermal Cells and Oenocytes

**FIGURE 2:**
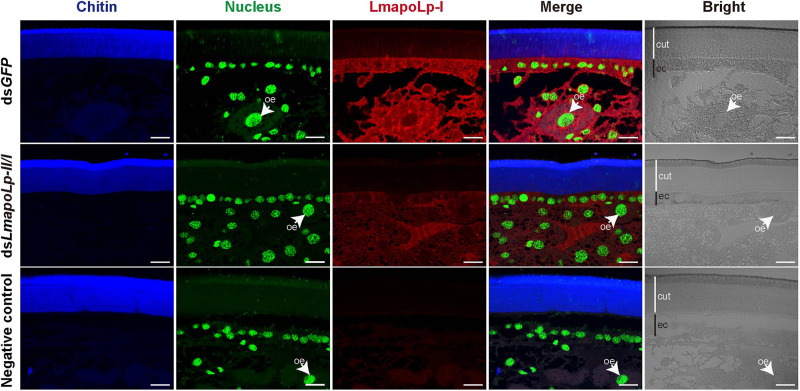
Localization of LmapoLp-I by immunohistochemistry. Chitin, nucleus and apolipophorins were detected by a blue, green, and red signal, respectively. A positive signal was detected in the epidermal cells and oenocytes of the ds*GFP*-injected nymphs. Whereas only a weak signal was detected in the ds*LmapoLp-II/I*-injected nymphs and almost no signal in the negative control. cut, cuticle; ec, epidermal cells; oe, oenocytes. Scale bar = 20 μm.

To scrutinize the biological function of LmapoLp-II/I, we sought to analyze its localization by immunodetection in the integument using a LmapoLp-I specific antibody (Figure 2). A positive signal was detected in the cytoplasm of epidermal cells and oenocytes in the ds*GFP*-injected control nymphs, whereas only a weak signal was detected in the ds*LmapoLp-II/I*-injected nymphs. This indicates that injection of ds*LmapoLp-II/I* suppressed LmapoLp-I protein accumulation. The localization of LmapoLp-I in the cytoplasm of epidermal cells and oenocytes further suggests that LmapoLp-I accumulated in those cells that express it.

### *LmapoLp-II/I* Is Essential for Locust Molting and Development

**FIGURE 3:**
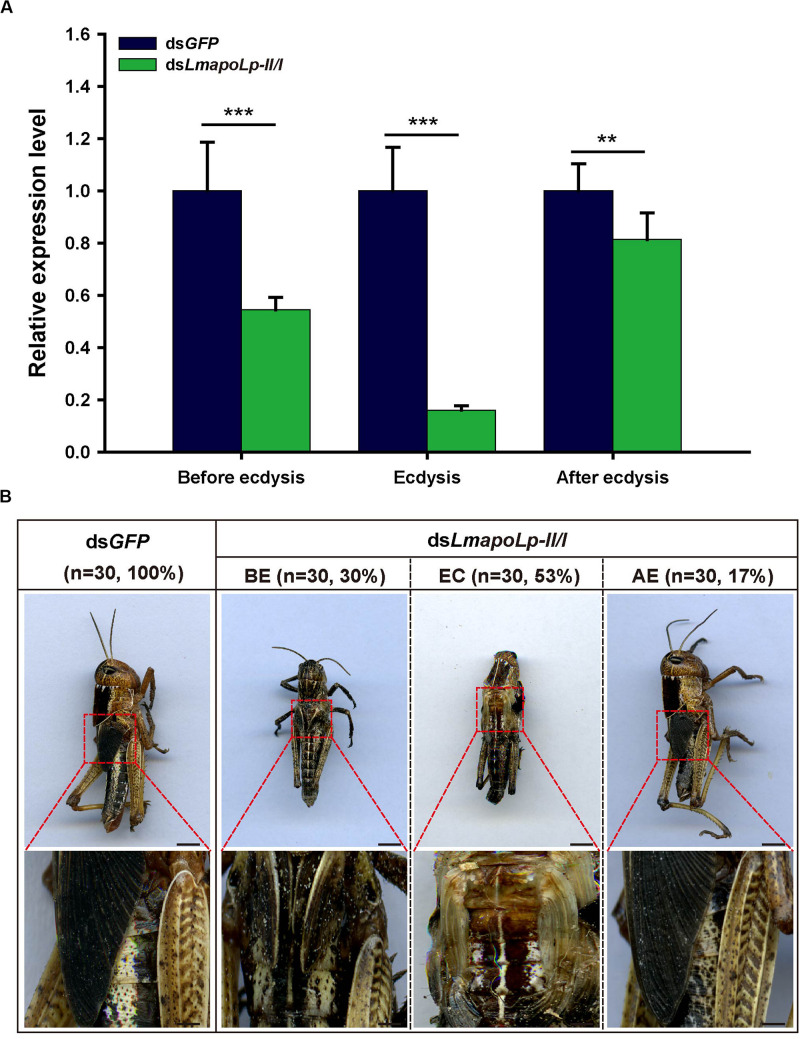
*LmapoLp-II/I* is required for locust molting. **(A)** Relative expression levels of *LmapoLp-II/I* in integuments after the ds*GFP* and ds*LmapoLp-II/I* injection was detected by RT-qPCR. *EF1-*α was used as the reference control. Data are shown as means ± SD from three independent experiments. Statistical significance was analyzed with Student’s *t*-test. Asterisks indicate significant differences (**P < 0.01; ***P < 0.001). **(B)** Phenotype of the nymphs after ds*GFP* and ds*LmapoLp-II/I* injection (*n* = 30). Nymphs injected with ds*GFP* were able to molt to fifth-instar normally. After injection of ds*LmapoLp-II/I*, 30% of nymphs died before ecdysis (BE), and 53% of nymphs died during ecdysis (EC). The remaining 17% of the ds*LmapoLp-II/I*-injected nymphs could molt to the fifth-instar nymphs, but died before molting to adults (after ecdysis, AE). Scale bar = 5 mm.

To investigate the role of *LmapoLp-II/I* during growth and development, dsRNAs directed against *LmapoLp-II/I* (ds*LmapoLp-II/I*) and *GFP* (ds*GFP*, control) transcripts were injected into fourth-instar nymphs (2-days-old, N4D2). Compared to the control, three different phenotypes at different stages (before ecdysis, during ecdysis, and after ecdysis) were observed in the ds*LmapoLp-II/I*-injected nymphs with a silencing efficiency of 45, 80, and 18%, respectively (Figure 3A). In ds*GFP*-injected control cohort, all nymphs molted normally to the next stage (fifth-instar nymphs), whereas 83% of the locusts injected with ds*LmapoLp-II/I* failed to molt to the next stage. These nymphs died either before ecdysis (30%) or during ecdysis (53%). The remaining 17% of the ds*LmapoLp-II/I*-injected nymphs molted to the next stage but died before molting to adults (Figure 3B). These results indicate that *LmapoLp-II/I* is essential for locust molting and development.

### Effects of *LmapoLp-II/I* RNAi on Neutral Lipid Accumulation in the Cuticle

**FIGURE 4:**
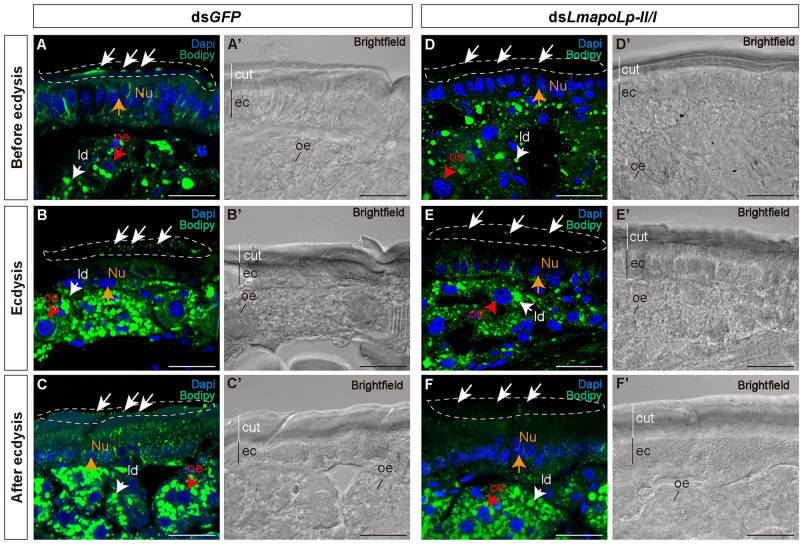
The effect of *LmapoLp-II/I* suppression on the content of neutral lipids in the cuticle. The cryosections (20 μm) of the abdominal cuticles from the nymphs after the injection of ds*GFP* **(A’–C’)** or ds*LmapoLp-II/I* **(D’–F’)** in 2-day-old fourth-instar nymphs were prepared and stained with Bodipy^505/515^ and DAPI to mark neutral lipid (green) and the nucleus (blue) of epidermal cells (ec), respectively. Compared to the ds*GFP*-injected group, the lipid droplets (ld) on the surface of the new cuticle (cut) were significantly reduced after injection with ds*LmapoLp-II/I* either before ecdysis **(D)**, during ecdysis **(E)** or after ecdysis **(F)** (white dashed box). While the Bodipy signal intensity seemed to be similar in the epidermis of control **(A,B)** and ds*LmapoLp-II/I* **(D,E)** nymphs before ecdysis and during ecdysis, it was clearly weaker in the epidermis of ds*LmapoLp-II/I* treated nymphs **(F)** than in the epidermis of control nymphs **(C)** after ecdysis. White arrows point to lipid droplets. Red arrows mark the oenocytes (oe). Orange arrows point to the nucleus (Nu). Scale bar = 20 μm.

To investigate the effect of *LmapoLp-II/I* on lipid accumulation in the cuticle, we performed histochemical experiments using fluorescent Bodipy^505/515^ that detects neutral lipids. Compared with the nymphs injected with ds*GFP* (Figures 4A–C,A’–C’), the green fluorescence on the surface of the post-molting cuticle was significantly reduced in the nymphs treated with ds*LmapoLp-II/I* (Figures 4D–F,D’–F’). The intensity of the Bodipy signal was similar in the epidermis of ds*GFP*- and *dsLmapoLp-II/I*-injected animals before and during ecdysis (Figures 4A,B,D,E), while it was obviously reduced in the epidermis of *dsLmapoLp-II/I*-injected nymphs after ecdysis (Figures 4C,F). The signal was comparable in the oenocytes and the fat body between ds*LmapoLp-II/I*- and ds*GFP*-injected nymphs, indicating that silencing of *LmapoLp-II/I* only affects the distribution and content of neutral lipids on the cuticle surface, but has no effect on the accumulation of internal neutral lipids.

### Analysis of Cuticular Hydrocarbons and Internal Hydrocarbons

**FIGURE 5:**
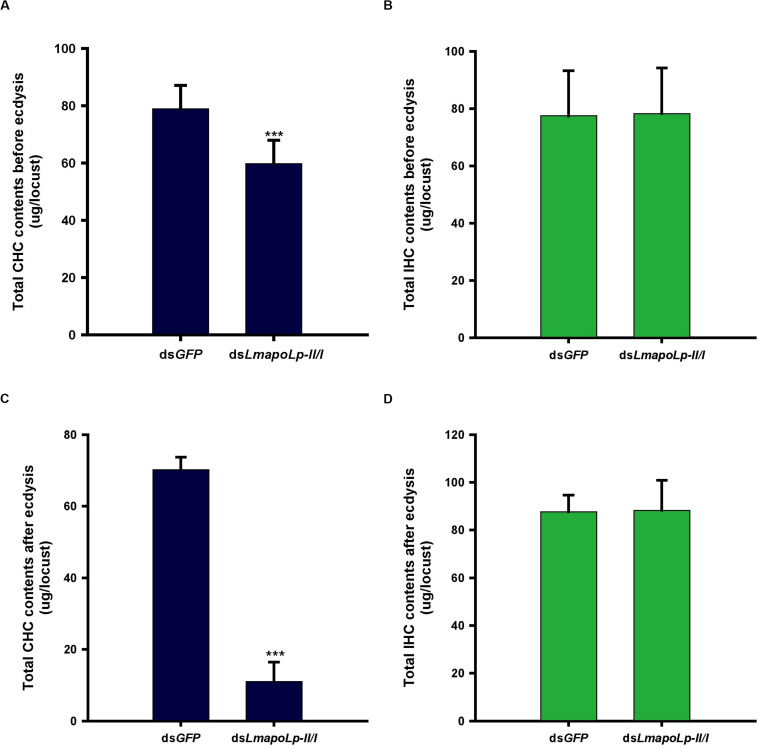
Effects of *LmapoLp-II/I* RNAi on the total CHC and IHC contents in *L. migratoria*. Total hydrocarbons contents extracted from the nymphs 48 h after ds*GFP* and ds*LmapoLp-II/I* injection (before ecdysis, **A,B**) and 5 h after ecdysis **(C,D)**, respectively. In ds*LmapoLp-II/I*-injected group, the total CHCs before ecdysis **(A)** and after ecdysis **(C)** decreased by 24 and 84% respectively, compared to the ds*GFP* control group. However, there was no significant difference in IHCs contents between the ds*LmapoLp-II/I-* and ds*GFP*-injected group nymphs **(B,D)**. The hydrocarbons are represented as micrograms (μg) per locust, and the data are shown as means ± SD. Statistical significance was analyzed with Student’s *t*-test. ***P < 0.001. *N* = 15–20 nymphs per treatment.

The cuticular lipids of locusts mainly consist of long chain fatty acids, methyl and ethyl esters and hydrocarbons that account for about 60% of cuticular lipids (Cerkowniak et al., 2013). The main components of the locust CHCs are *n*-alkanes and methyl alkanes (Pedrini et al., 2007). To detect the effects of *LmapoLp-II/I* RNAi on cuticular lipid deposition, the CHCs and IHCs from ds*GFP*- and ds*LmapoLp-II/I*-injected nymphs were extracted before ecdysis and after ecdysis and analyzed by GC-MS. The results showed that, compared to the ds*GFP*-injected group, the total alkane content of CHC was significantly decreased by 24% in the ds*LmapoLp-II/I*-injected nymphs before ecdysis (Figure 5A), and by 84% after ecdysis (Figure 5C). The extent of reduction after ecdysis is much higher than that of before ecdysis. This reduction was systematically affecting almost all surface-cuticular alkanes from C25 to C33 and methyl alkanes from MeC25 to MeC31 (Supplementary Figure S2). However, there were no significant differences of alkane contents in the IHC extracts between the ds*LmapoLp-II/I*- and ds*GFP*-injected groups (Figures 5B,D and Supplementary Figure S3) except for two methyl alkanes, 3-MeC27 and 12-MeC30, that were slightly decreased in the ds*LmapoLp-II/I*-injected group after ecdysis (Supplementary Figure S3D). Because the CHC amounts depend on the lipid synthesis process, we monitored the expression levels of genes related to lipid synthesis after silencing of *LmapoLp-II/I*. The results showed that, compared to the control, the mRNA levels of *LmFAS1-3*, *LmELO1*, and *LmELO7* were significantly decreased after deficiency of *LmapoLp-II/I* (Supplementary Figure S4). This effect may reduce the synthesis of hydrocarbons and subsequently result in reduction of CHCs in ds*LmapoLp-II/I*-injected animals. Taken together, we conclude that *LmapoLp-II/I* is involved in the deposition of CHCs, but has almost no effect on IHCs.

### *LmapoLp-II/I* RNAi Affected the Sensitivity of Locusts to Desiccation

**FIGURE 6:**
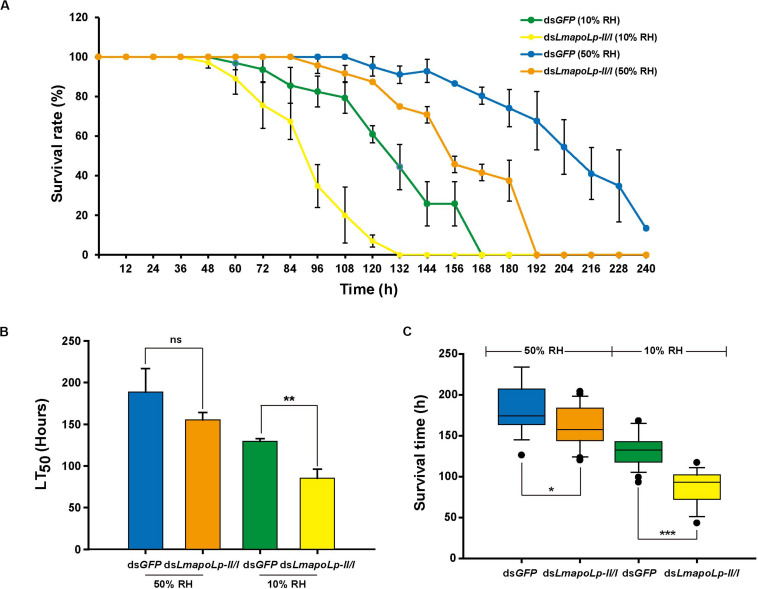
*LmapoLp-II/I* is essential for desiccation resistance of *L. migratoria*. **(A)** Temporal profile of survival rate of ds*GFP*- and ds*LmapoLp-II/I*-injected nymphs at different stages after desiccation treatment. Green line: ds*GFP*-injected nymphs at 10% relative humidity. Yellow line: ds*LmapoLp-II/I*-injected nymphs at 10% relative humidity. Blue line: ds*GFP*-injected nymphs at 50% relative humidity. Orange line: ds*LmapoLp-II/I*-injected nymphs at 50% relative humidity. **(B)** The median lethal time (LT_50_) calculated from the time-dependent survival rates. **(C)** Survival time of ds*GFP*- and ds*LmapoLp-II/I*-injected nymphs after desiccation treatment. All data are reported as means ± SD of three independent biological replications. Statistical significance was analyzed with Student’s *t*-test. Asterisks indicate significant differences (*P < 0.05; **P < 0.01; ***P < 0.001).

The water-retention capacity of the cuticle depends on the lipids deposited on its surface (Gibbs, 1998; Howard and Blomquist, 2005). In order to determine whether knockdown of *LmapoLp-II/I* affects the adaptability of *L. migratoria* to drought, a desiccation experiment was conducted with nymphs at 24 h after ds*LmapoLp-II/I* injection. The results showed that the survival rate of the nymphs injected with ds*LmapoLp-II/I* declined significantly compared to the ds*GFP*-treated control after desiccation treatment (10% RH) (*P* = 7.94e-7, Figure 6A and Supplementary Figure S5), the median lethal time (LT_50_ ± SEM) strongly decreased from 129.7 ± 2.9 h to 85.3 ± 10.8 h after injection of ds*LmapoLp-II/I* (Figure 6B). Similar results were obtained after correction of the survival rate by the subtraction of the values determined at 50% RH from those determined at 10% RH (Supplementary Figure S6). In addition, the survival time of ds*LmapoLp-II/I*-treated nymphs decreased by 33% compared with the ds*GFP* control nymphs (Figure 6C). However, there was no significant difference in LT_50_ between ds*LmapoLp-II/I-* and ds*GFP*-injected locusts at 50% RH, but the survival time decreased by 11% (Figures 6B,C). These results indicate that *LmapoLp-II/I* is able to modulate desiccation resistance of *L. migratoria*.

### Suppression of *LmapoLp-II/I* Enhanced Cuticle Penetration and Increases Its Susceptibility to Insecticides

**FIGURE 7:**
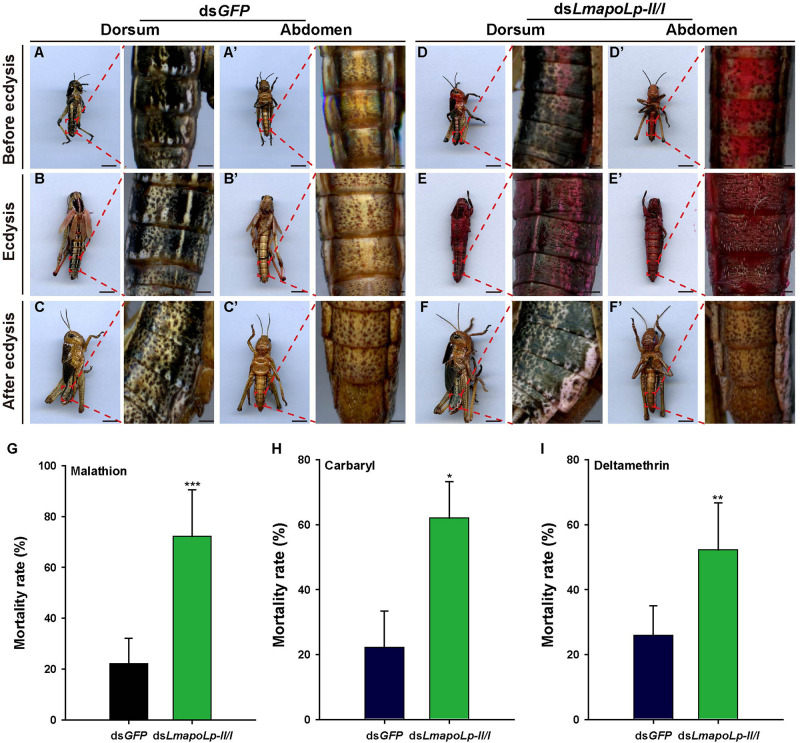
Suppression of *LmapoLp-II/I* enhances cuticle permeability and susceptibility to insecticides. Dorsal and ventral views of the nymphs with different phenotypes (before ecdysis, during ecdysis, and after ecdysis) after ds*GFP* and ds*LmapoLp-II/I* injection in 2-day-old fourth-instar nymphs are presented. Nymphs injected with ds*GFP* **(A–C,A’–C’)** were not stained by Eosin Y at 45°C, while both the dorsal and ventral sides of ds*LmapoLp-II/I*-injected **(D–F,D’–F’)** group nymphs were stained by Eosin Y. **(G–I)** Mortality rate of the third-instar nymphs injected with ds*GFP* (blue black column) or ds*LmapoLp-II/I* (green column) after insecticide exposure for 24 h. Asterisks indicate significant differences (*P < 0.05; **P < 0.01; ***P < 0.001). Data is represented as means ± SD of the five biological repeats. Statistical significance was analyzed with Student’s *t*-test.

To investigate whether suppression of *LmapoLp-II/I* affects the inward barrier function of the cuticle, Eosin Y staining was applied to examine cuticle permeability. As shown in Figure 7, Eosin Y did not penetrate into the cuticle of ds*GFP*-injected nymphs after incubation for 30 min at 45°C at any stage of ecdysis (Figures 7A–C,A’–C’). In contrast, the dorsum and abdomen of ds*LmapoLp-II/I*-injected nymphs before ecdysis and during ecdysis were stained by Eosin Y under the same conditions (Figures 7D,E,D’,E’). Nymphs that did not die immediately after ecdysis were stained slightly by Eosin Y (Figures 7F,F’). Together, these data suggested that suppression of *LmapoLp-II/I* enhances the permeability of the cuticle.

To further investigate the inward barrier function of locusts with dysfunction of *LmapoLp-II/I*, three types of insecticides (malathion, carbaryl and deltamethrin) were applied to ds*LmapoLp-II/I*-treated third-instar nymphs. Our results showed that knockdown of *LmapoLp-II/I* significantly increased sensitivity of nymphs to the three insecticides with a mortality of 72.2, 62, and 52.2% compared to 22.2, 22.2, and 25.9% in the control group, respectively (Figures 7G–I). These results indicate that silencing of *LmapoLp-II/I* significantly increases the susceptibility of *L. migratoria* to insecticides by enhancing the permeability of the cuticle.

## Discussion

ApoLps were characterized for their roles in lipid transportation between organs such as fat body, guts, wing discs, and oocytes (Benoit et al., 2011; Palm et al., 2012). They have also been implicated in innate immune responses (Zdybicka-Barabas and Cytryńska, 2013; Wen et al., 2017). ApoLp-II/I arise from a common precursor protein apoLp, which are invariably present in a 1:1 molar ratio (Weers et al., 1993). In the present study, by using the orthopteran insect *L. migratoria* as a model animal, we analyzed the characteristics and functions of *LmapoLp-II/I* in the deposition of lipids on the cuticle surface. To address different aspects of *LmapoLp-II/I* function, different nymphal stages were chosen depending the suitability of the size of the nymph for the respective experiment. Most experiments including immunohistochemistry, RT-qPCR analyses and mass spectrometry were performed on 4th and 5th instar nymphs whereas for insecticides tests third instar nymphs were used. We believe that the data are transferable between stages and will test this possibility in the next future.

### *LmapoLp-II/I* Is Conserved Among Insect Species

Many apoLp-II/I proteins have been identified from different insect species including ApapoLp-II/I, BmapoLp-II/I, DmapoLp-II/I, and LmapoLp-II/I. They all contain LPD_N, DUF1943, DUF1081, and VWD domains (Bogerd et al., 2000; Hanada et al., 2011; Wen et al., 2017), indicating that apoLp-II/I proteins are conserved among insect species. As reported by Bogerd et al. (2000), the transcript of *LmapoLp-II/I* was mainly detected in the fat body and in the brain. In our work, besides in the fat body, we also found that *LmapoLp-II/I* was predominantly expressed in the integument, but showed low expression levels in the other tested tissues. Similarly, the transcript of *apoLp-II/I* in *Antheraea pernyi* was mainly expressed in the fat body and the integument, but weakly in other tissues (Wen et al., 2017). Thus, the fat body and the integument might be the main tissues expressing *apoLp-II/I* in insects. In addition, using a LmapoLp-I antibody, we found that the LmapoLp-I protein is located in epidermal cells and oenocytes. This finding suggests that LmapoLp-II/I is a carrier between oenocytes and epidermal cells in the integument. It is well possible that the actual sites of *apoLp-II/I* expression in the integument are not the epidermal cells themselves but the associated oenocytes that are hepatocyte-like cells involved in lipid production and circulation in insects (Schal et al., 1998; Gutierrez et al., 2007; Makki et al., 2014). Thus, both the expression pattern of *apoLp-II/I* and its encoded protein are conserved among insect species.

### *LmapoLp-II/I* Is Required for Deposition of Cuticular Lipids

The transport of cuticular lipids in insects mainly occurs during molting, and a number of lipids are secreted by oenocytes and transported to the cuticle for deposition immediately after formation of the new cuticle (Qiu et al., 2012; Flaven-Pouchon et al., 2016). The expression profiles of *LmapoLp-II/I* during developmental stages showed that the transcript levels of *LmapoLp-II/I* increased after ecdysis and reached a peak on day 3 and 4 before decreasing gradually. In the fruit fly *D. melanogaster*, initial studies have shown that *apoLp-II/I*-RNAi caused accumulation of neutral lipids in the gut and reduction of lipid droplets in fat body cells and imaginal discs, suggesting that apoLp-II/I is required for the delivery of lipids from the gut to other tissues (Panakova et al., 2005). Subsequent studies confirmed that apoLp-II/I is an important lipid carrier in the hemolymph, and that apoLp-II/I produced in the fat body is recruited to the gut to mobilize lipids taken up by the midgut (Palm et al., 2012). By Bodipy staining, we showed that the amounts of surface lipids in the abdomen of the ds*LmapoLp-II/I*-injected nymphs were significantly reduced compared to those of ds*GFP*-injected nymphs. We confirmed this observation by GC-MS analyses of CHC amounts including normal and methyl-branched alkanes in control and ds*LmapoLp-II/I*-injected nymphs. The reduction of CHC amounts before ecdysis was modest, conceivably because the bulk of hydrocarbons had been synthesized and transported to the cuticle before ds*LmapoLp-II/I* injection. By contrast, the reduction of CHC amounts after ecdysis was strong in ds*LmapoLp-II/I*-treated nymphs. Obviously, LmapoLp-II/I is needed for the deposition of large amounts of CHCs on the surface of the newly formed cuticle. Incidentally, the process of CHC reconstitution after ecdysis involving LmapoLp-II/I seems to be rather fast in the locust. Indeed, the amounts of CHCs before and immediately after ecdysis are similar. By contrast, in the fruit fly *D. melanogaster*, the CHC levels reach their peak several days after eclosion (Ferveur et al., 1997; Savarit and Ferveur, 2002). An interesting observation was that the levels of most of the IHCs tested were stable in the ds*LmapoLp-II/I*-injected group before and after ecdysis. The exceptions were the levels of 3-MeC27 and 12-MeC30 that decreased by 27 and 35%, respectively, after ecdysis. Overall, we reckon that a possible explanation for the stability of the levels of most of the IHCs is feedback regulation at the transcriptional level. The concentrations of hydrocarbons inside the cell presumably control the abundance of transcripts of enzymes involved in hydrocarbon production. Indeed, this kind of feedback regulation is recurrent in lipid metabolism (Jacquemyn et al., 2017; Tsai et al., 2019). This explanation obviously does not apply to the levels of 3-MeC27 and 12-MeC30. Identification and characterization of the enzymes responsible for 3-MeC27 and 12-MeC30 production should shed light on this problem. In summary, our data indicate that *LmapoLp-II/I* expressed in the fat body and the integument is needed for CHC deposition on the surface of the locust cuticle during molting.

### *LmapoLp-II/I* Is Essential for the Barrier Function of the Cuticle

In this study, after silencing of *LmapoLp-II/I* by dsRNA injection, about 83% of the nymphs failed to molt and died. As a protein involved in the fundamental process of lipid trafficking, there may be many reasons of ds*LmapoLp-II/I*-induced lethality. Conceivably, a major reason for ds*LmapoLp-II/I*-induced lethality is a weakened cuticle barrier function due to reduction of surface lipids. It is commonly agreed that surface lipids including CHCs are crucial components of the cuticle barrier against desiccation and xenobiotics penetration (Howard and Blomquist, 2005; Balabanidou et al., 2016, 2019; Wen et al., 2016). Indeed, we found that ds*LmapoLp-II/I*-injected nymphs are sensitive to low air humidity, insecticide application and Eosin Y penetration. Thus, *LmapoLp-II/I* contributes to the construction and function of both inward and outward cuticular barriers.

Our findings, however, suggest an unexpected complexity in barrier formation in the locust. Just after ecdysis, CHC levels strongly dropped in ds*LmapoLp-II/I*-injected nymphs, whereas, contra-intuitively, Eosin Y penetration was weaker at this stage compared to the situation before ecdysis. In a simple view, there is, hence, a contradiction to the assumption that CHCs constitute an inward barrier. We speculate that compensatory mechanisms in survived nymphs alleviate failure in CHC deposition. A possible compensatory mechanisms is melanization that according to the melanism-desiccation hypothesis by Kalmus (1941) prevents desiccation. In a recent work, it was shown that the melanization degree of the fruit fly cuticle correlates with CHC amounts suggesting the melanization and CHC production pathways interact (Massey et al., 2019). In line with this argument, CHC reduction in ds*LmapoLp-II/I*-injected nymphs may induce cuticle melanization and by consequence increase cuticle barrier function. To substantiate this possibility, the melanization degree of the cuticle of ds*LmapoLp-II/I*-injected nymphs should be determined.

With this work, we add LmapoLp-II/I to a row or cascade of proteins and enzymes that have been demonstrated to be needed for CHC production, transport and deposition in the locust. In oenocytes or the fat body, fatty acids are decarbonylated to produce CHCs by LmCYP4G102 (Yu Z. et al., 2016). Subsequently, LmapoLp-II/I delivers these CHCs to the epidermal cells where they are transported to the surface probably by the ABC transporter ABCH-9C (Yu et al., 2017). Taking work done in other species into account, we are close to a comprehensive view on these processes in insects. The fatty acid synthesis initiating enzymes acetyl-CoA Carboxylase (ACC) and fatty acid synthase 3 (FASN3) and the lipophorin receptors LpR1 and LpR2 in *D. melanogaster*, for instance, have been reported to be essential for CHC production or trafficking and against water-loss (Wicker-Thomas et al., 2015). In addition, in the brown plant hopper *Nilaparvata lugens*, some fatty acid elongases (ELOs) and reductases (FARs) have been shown to be crucial for correct CHC amounts and desiccation resistance in this rice pest (Li et al., 2019, 2020). Taken together, these proteins needed for CHC production or trafficking are crucial for water-loss and insecticide-penetration, thereby modulate the ecological adaptability of insects. In conclusion, the susceptibility of this pathway to perturbation, assigns its proteins and enzymes as perfect targets for intelligent pest control in the future.

## Data Availability Statement

The datasets presented in this study can be found in online repositories. The names of the repository/repositories and accession number(s) can be found in the article/Supplementary Material.

## Ethics Statement

Ethical review and approval was not required for the animal study because no vertebrates and higher invertebrates studies and no potentially identifiable human images or data are presented in this study. The locusts used in this study are lower invertebrates and are not regulated animals (i.e., all live vertebrates and higher invertebrates) which are exempt from ethics approval.

## Author Contributions

JZZ conceived and designed the study. YZ, HG, and YY performed the experiments. YZ, WL, XZ, ZY, and BM analyzed the data. YZ, WL, and XZ drafted the manuscript and prepared the figures. All authors interpreted the results, edited and revised the manuscript, and approved the final version of the manuscript.

## Conflict of Interest

The authors declare that the research was conducted in the absence of any commercial or financial relationships that could be construed as a potential conflict of interest.

## Footnotes

1 http://www.expasy.org/tools/dna.html

2 http://smart.embl.de/

3 http://www.expasy.org

4 http://www.dkfz.de/signaling/e-rnai3//
