# Supplementary material for: Apolipophorin-II/I Contributes to Cuticular Hydrocarbon Transport and Cuticle Barrier Construction in Locusta migratoria
Source: Front Physiol. 2020 Jul 8;11:790. doi: 10.3389/fphys.2020.00790 (PMC7360829; doi:10.3389/fphys.2020.00790)
Supplement: Supplementary file 1 [file Table_1.DOC]

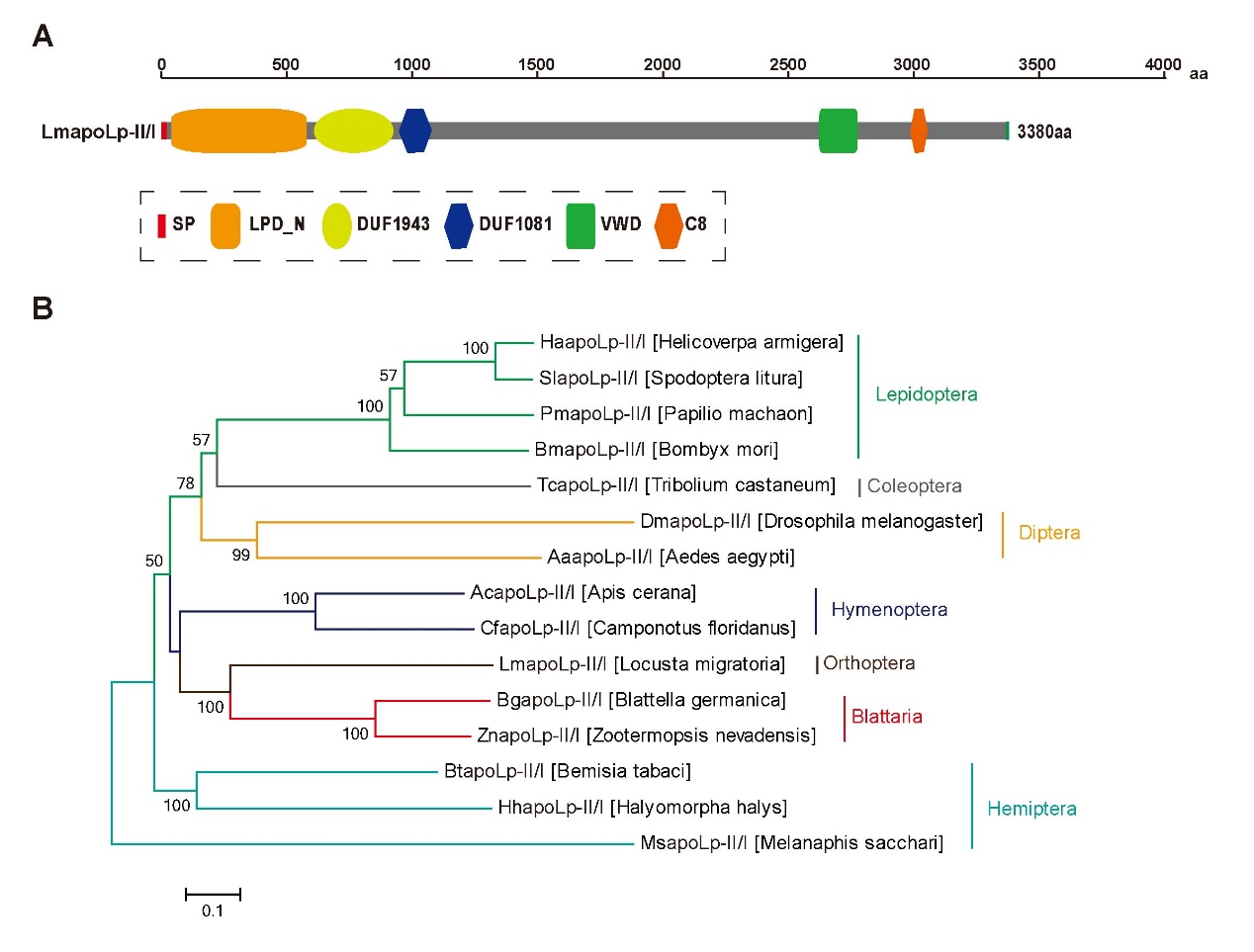


**FIGURE S1｜**Structure and phylogenetic analysis of LmapoLp-II/I. **(A)** Schematic diagram of deduced domains of LmapoLp-II/I. The red rectangle represents the signal peptide (SP). The orange and green boxes represent the lipoprotein N-terminal domain (LPD-N) and the von Willebrand factor (vWF) type D domain (VWD), respectively. The yellow oval represents DUF1943. The blue and orange polygons represent DUF1081 and C8, respectively. **(B)** Phylogenetic analysis of homologous proteins from different insect species using the Neighbor-Joining method of MEGA 5.1 software. Bootstrap support was based on 1000 resembled data sets.


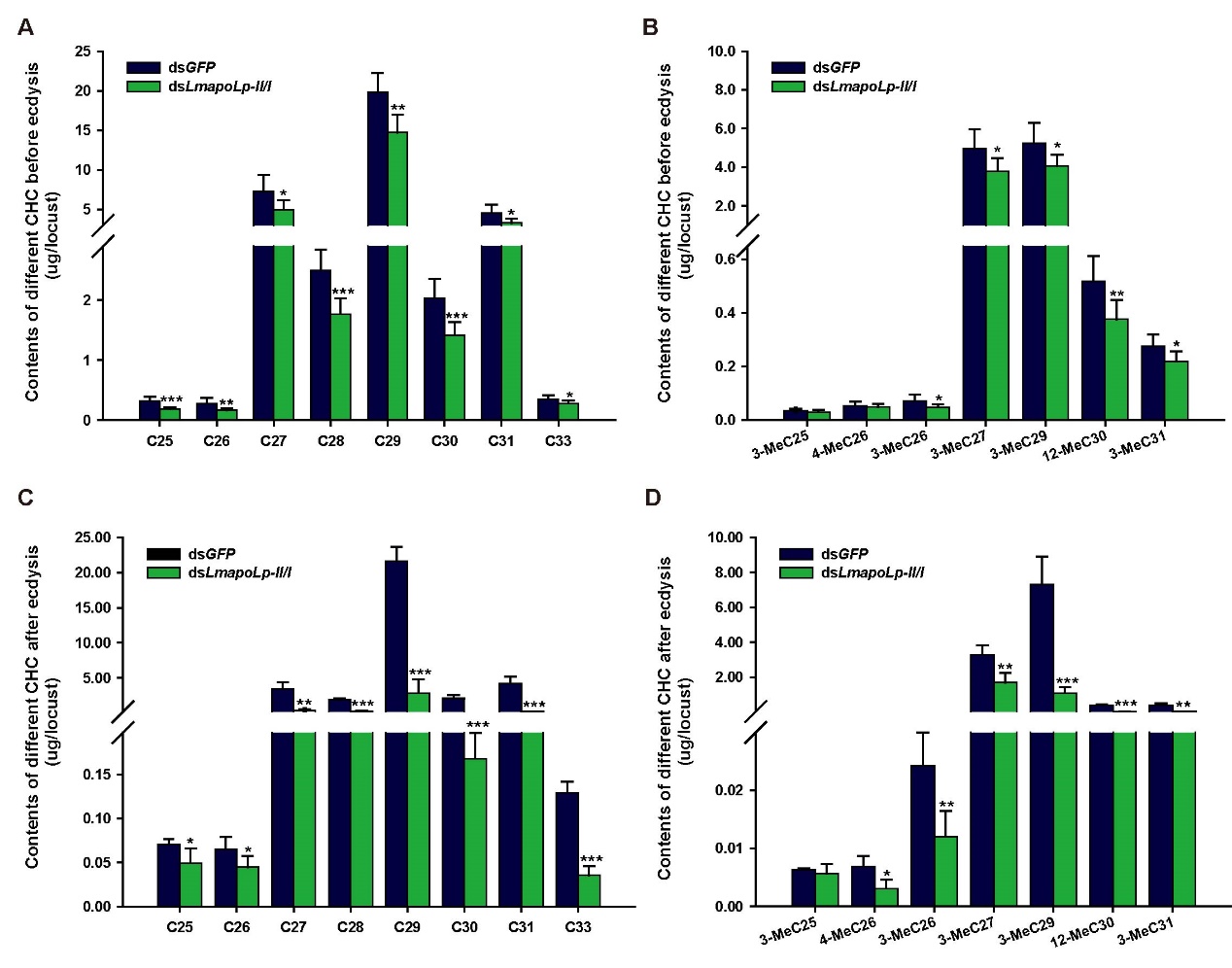


**FIGURE S2｜**Effects of *LmapoLp-II/I* RNAi on the CHC contents of the nymphs. **(A, C)** Contents of unbranched alkanes of different lengths (from C25–C33). **(B, D)** Contents of methyl alkanes of different lengths (from MeC25 to MeC31). In ds*LmapoLp-II/I*-injected group, the CHCs contents decreased significantly either before ecdysis **(A-B)** or after ecdysis **(C-D)**, compared to the ds*GFP* control animals. The hydrocarbons are represented as micrograms (μg) per locust, and the data are shown as means ± SD. Statistical significance was analyzed with Student’s *t*-test. *, *P* < 0.05; **, *P* < 0.01; ***, *P* < 0.001. N = 15-20 nymphs per treatment.


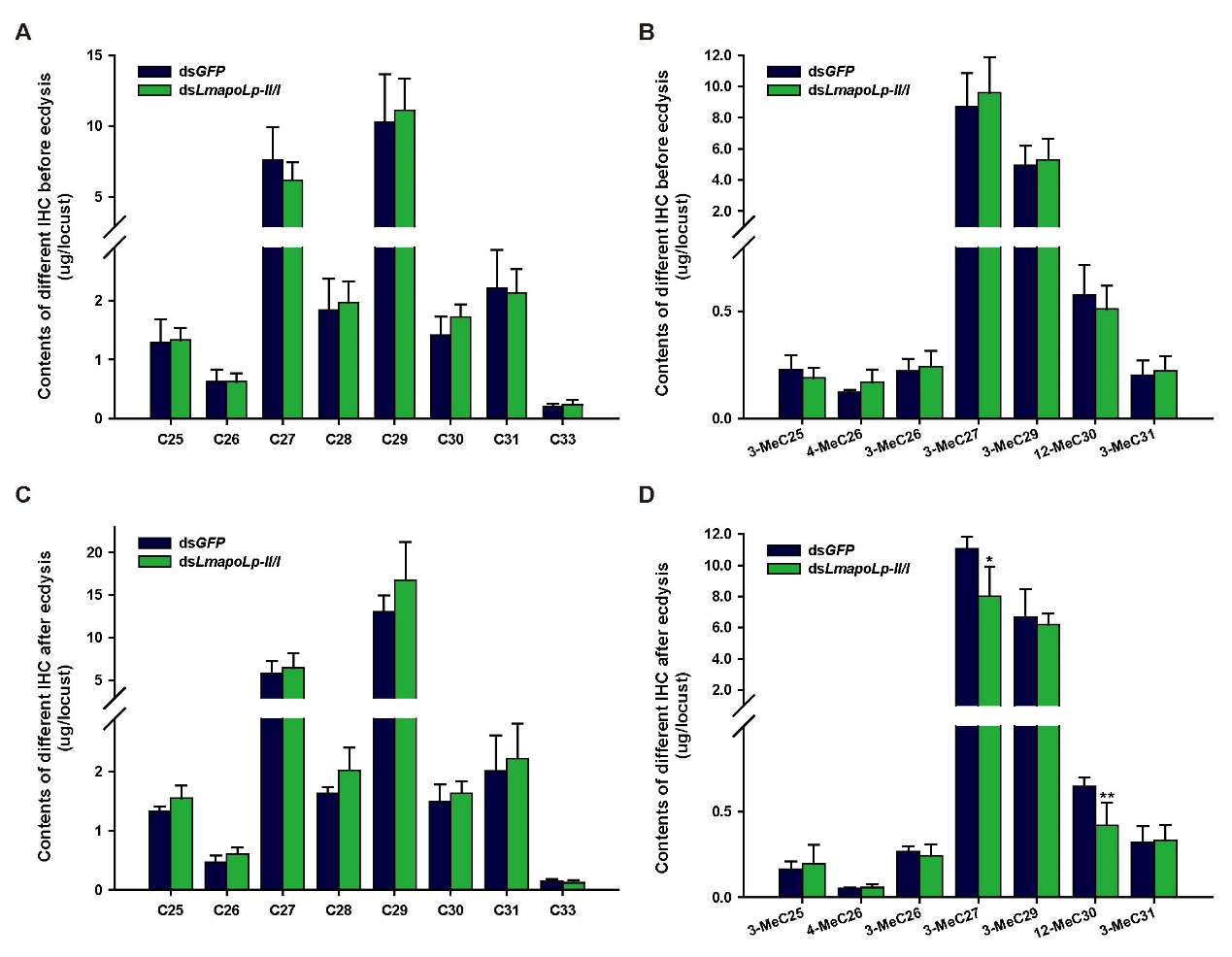


**FIGURE S3｜**Effects of *LmapoLp-II/I* RNAi on the IHC contents of the nymphs. **(A, C)** Contents of unbranched alkanes of different lengths (from C25–C33). **(B, D)** Contents of methyl alkanes of different lengths (from MeC25 to MeC31). There was no significant difference in IHCs contents between the ds*LmapoLp-II/I*- and ds*GFP*-injected group nymphs neither before ecdysis **(A-B)** nor after ecdysis **(C-D)**, except for C27 and C30 methyl alkanes that were reduced weakly after ecdysis **(D)**. The hydrocarbons are represented as micrograms (μg) per locust, and the data are shown as means ± SD. Statistical significance was analyzed with Student’s *t*-test. *, *P* < 0.05; **, *P* < 0.01. N = 15-20 nymphs per treatment.


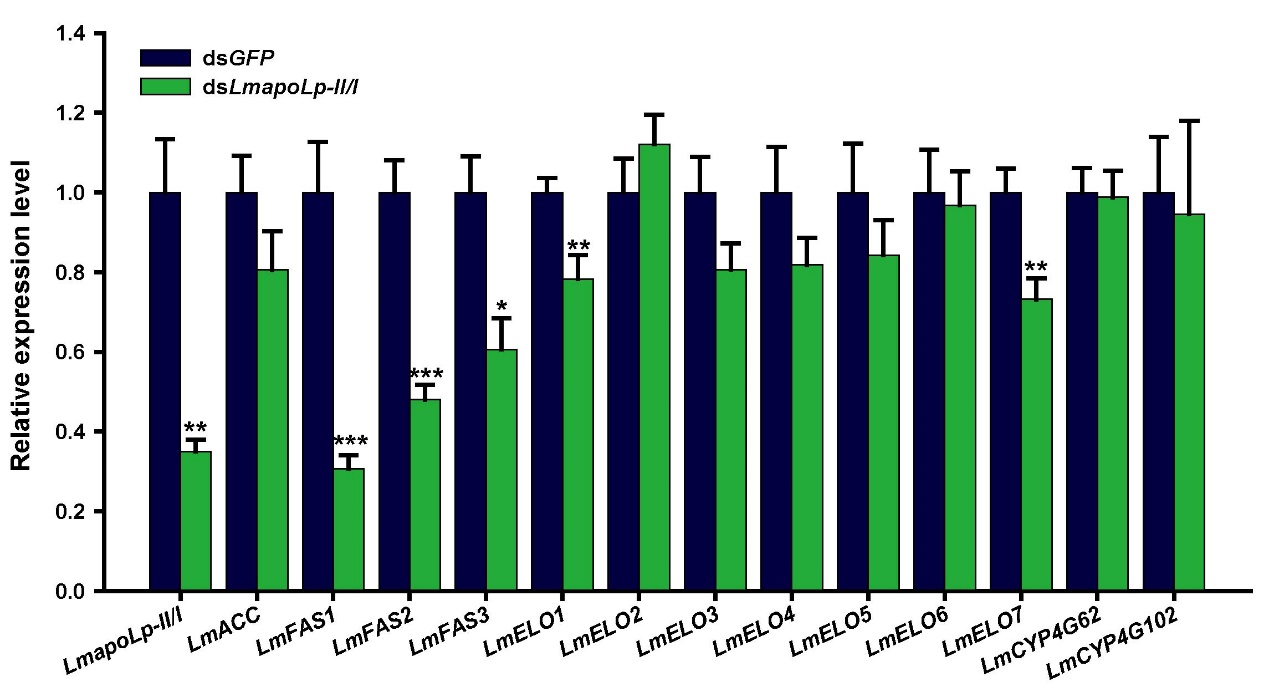


**FIGURE S4｜***LmapoLp-II/I* affects on the expression of lipid synthesis related genes. The expression of lipid synthesis related genes was monitored by RT-qPCR after silencing of *LmapoLp-II/I*. *EF1-α* was used as the reference control gene. The silencing of *LmapoLp-II/I* resulted in decrease of *LmFAS1-3*, *LmELO1* and *LmELO7*. All data are reported as means ± SD of four independent biological replications. Statistical significance was analyzed with Student’s *t*-test (*, *P* < 0.05; **, *P* < 0.01; ***, *P* < 0.001).


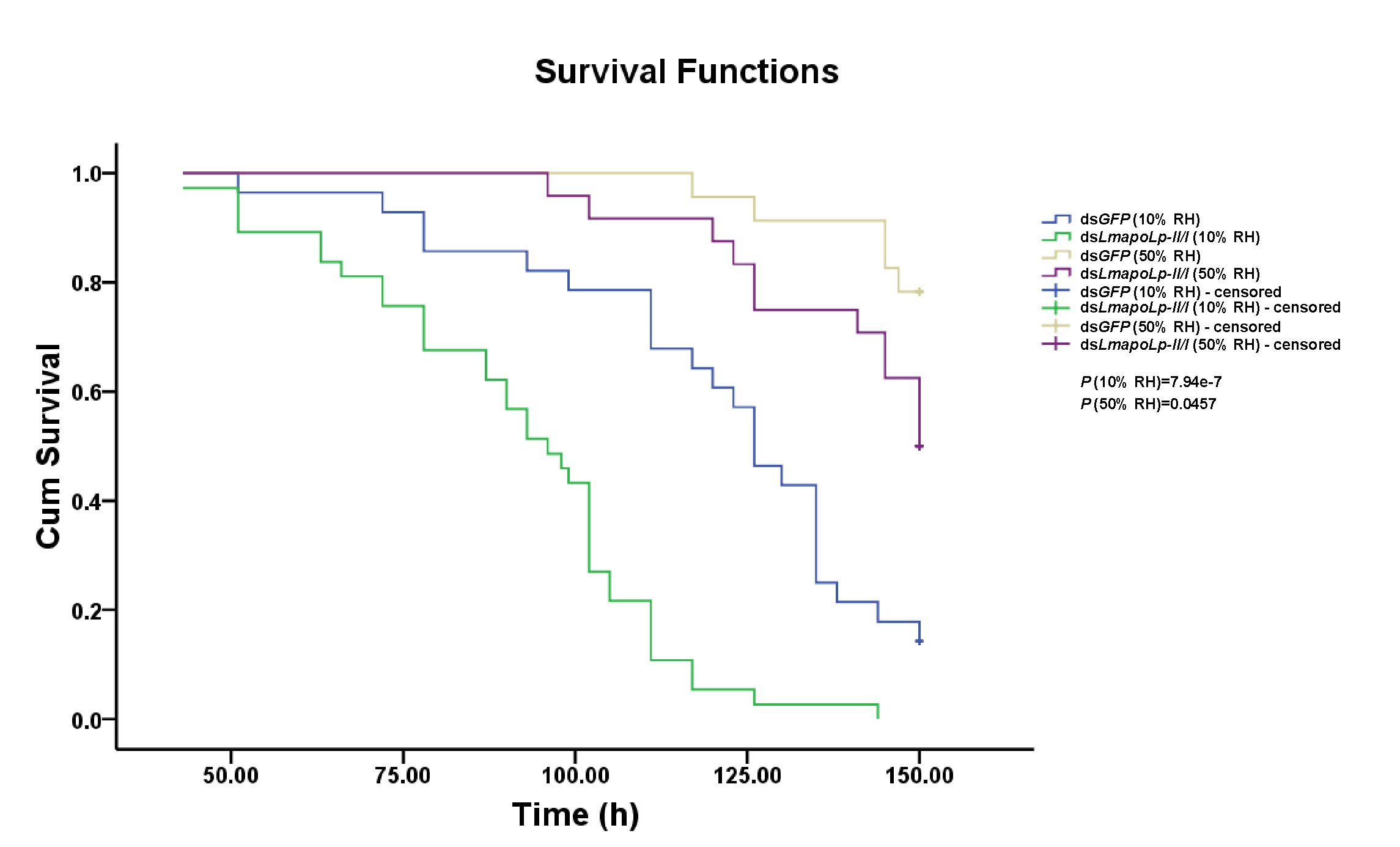


**FIGURE S5｜**Kaplan-Meier survival curves for ds*GFP* and ds*LmapoLp-II/I*-injected nymphs after desiccation treatment. Statistical significance was analyzed with log-rank test.


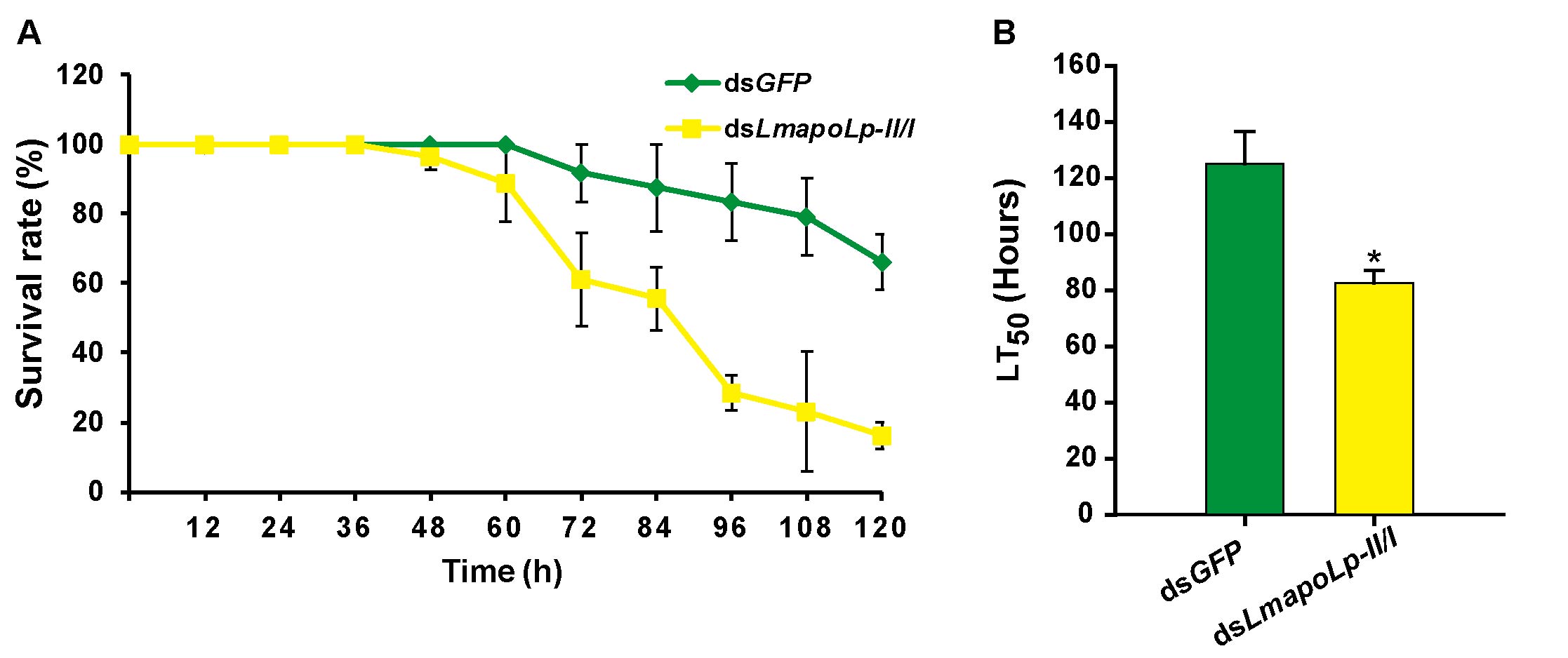


**FIGURE S6｜**Corrected results of desiccation experiment. **(A)** Temporal profile of survival rate of ds*GFP*- and ds*LmapoLp-II/I*-injected nymphs at different stages after desiccation treatment. Green line: ds*GFP*-injected nymphs at 10% relative humidity. Yellow line: ds*LmapoLp-II/I*-injected nymphs at 10% relative humidity. **(B)** The median lethal time (LT_50_) calculated from the time-dependent survival rates. The data were calculated by substracting the effect at 50% RH from the effect at 10% RH. Statistical significance was analyzed with Student’s *t*-test. Asterisks indicate significant differences (*, *P* < 0.05).

**TABLE S1｜Species and GenBank accession no. for Phylogenetic tree used in this study**

|  | **Species** | **Gene name** | **GenBank accession no.** |
| --- | --- | --- | --- |
|  | *Helicoverpa armigera*  *Spodoptera litura*  *Papilio machaon*  *Bombyx mori*  *Tribolium castaneum*  *Drosophila melanogaster*  *Aedes aegypti*  *Apis cerana*  *Camponotus floridanus*  *Locusta migratoria*  *Blattella germanica*  *Zootermopsis nevadensis*  *Bemisia tabaci*  *Halyomorpha halys*  *Melanaphis sacchari* | *HaapoLp-II/I*  *SlapoLp-II/I*  *PmapoLp-II/I*  *BmapoLp-II/I*  *TcapoLp-II/I*  *DmapoLp-II/I*  *AaapoLp-II/I*  *AcapoLp-II/I*  *CfapoLp-II/I*  *LmapoLp-II/I*  *BgapoLp-II/I*  *ZnapoLp-II/I*  *BtapoLp-II/I*  *HhapoLp-II/I*  *MsapoLp-II/I* | XP_021197525.1  XP_022837130.1  KPJ14603.1  XP_004926642.1  XP_015836415.1  AHN58222.1  XP_021707150.1  XP_016908190.1  XP_011252932.2  CAB51918.2  PSN46736.1  XP_021922987.1  XP_018904056.1  XP_014274514.1  XP_025197370.1 |

**TABLE S2｜Primers for PCR amplification and dsRNA synthesis.**

| **Application of primers** | **Primer names** | **Sequence of primers (5’-3’)** | **Products (bp)** |
| --- | --- | --- | --- |
| dsRNA synthesis | ds*LmapoLp-II/I*-F  ds*LmapoLp-II/I*-R | taatacgactcactatagggCTCCAAGCAGAAGGTCGTG  taatacgactcactatagggCAGGCTGAGATGAGAATGT | 460 |
|  | ds*GFP*-F | taatacgactcactatagggGTGGAGAGGGTGAAGG | 571 |
|  | ds*GFP*-R | taatacgactcactatagggGGGCAGATTGTGTGGAC |  |
| RT-qPCR analysis | *LmapoLp-II/I*-F  *LmapoLp-II/I*-R | AGCGATTTCATCCGGTGGC  GGTGTATGTCTGTCCCTTTT | 191 |
|  | *EF1α*-F  *EF1α*-R  *LmELO1*-F  *LmELO1*-R  *LmELO2*-F  *LmELO2*-R  *LmELO3*-F  *LmELO3*-R  *LmELO4*-F  *LmELO4*-R  *LmELO5*-F  *LmELO5*-R  *LmELO6*-F  *LmELO6*-R  *LmELO7*-F  *LmELO7*-R  *LmFAS1*-F  *LmFAS1*-R  *LmFAS2*-F  *LmFAS2*-R  *LmFAS3*-F  *LmFAS3*-R  *LmACC*-F  *LmACC*-R  *LmCYP4G62*-F  *LmCYP4G62*-R  *LmCYP4G102*-F  *LmCYP4G102*-R | AGCCCAGGAGATGGGTAAAG  CTCTGTGGCCTGGAGCATC  ACATCAGTCGGAGAAGTCG  TCAAGTCCTGGAAATCGTC  AGGAACAGGGCTTTCATCG  ACGCATCCACCACAGTCAG  TCTTGACACTGCCAAATGC  TTTCTCCTGAACGGACTGA  TTTTGCTGTGCGGTGATTC  TATGAGCGTGGGCATTGGA  TTACGATGCCTACAACTACT  CCAGACAATGAGCAAATAC  CTGCAATGACTCTGGTCCGATAA  GCGCTGGTCACTCCTGTTGTC  CGGCGGCGATAAGAAGAAC  CGGAAGCGAAATGAACTGC  TGTTGAAGTGCCTGGAGAT  GTGGGTTTGATGAAGGAGTTT  TTAGTGGAAAGGGAGGC  CCATACAAGGGTCAGGT  TCACTGGAACGGAAACGAAA  CCATAGCAAATGCAAAGGGT  GTGTGTTGGAGCCAGAAGGAAT  CACTTGGAAGGTTAGGAGAGGA  TAATACATTACTGGCAACAGAACAC  TTGCGGACACCAAATAGATAG  TACTTCGTCTACTACAAGCTGCAGC  CGCTCGAAAATCTCGTCATT | 155  162  132  101  166  127  133  115  125  140  195  132  112  131 |
